# Polyetheretherketone bioactivity induced by farringtonite

**DOI:** 10.1038/s41598-024-61941-3

**Published:** 2024-05-28

**Authors:** Martina Martínková, Lucie Zárybnická, Alberto Viani, Michael Killinger, Petra Mácová, Tomáš Sedláček, Veronika Oralová, Karel Klepárník, Petr Humpolíček

**Affiliations:** 1 Centre of Polymer Systems, Tomas Bata University in Zlín, tř. Tomáše Bati 5678, 760 01 Zlín, Czech Republic; 2 Institute of Theoretical and Applied Mechanics of the Czech Academy of Sciences, Centre Telč, Prosecká 809/76, 190 00 Praha 9, Czech Republic; 3 Dipartimento di Scienze Chimiche e Geologiche, Università degli Studi di Modena e Reggio Emilia, Via Campi, 103, 41125 Modena, Italy; 4 Department of Bioanalytical Instrumentation, Institute of Analytical Chemistry, Czech Academy of Sciences, Veveří 97, 602 00 Brno, Czech Republic; 5 Laboratory of Odontogenesis and Osteogenesis, Institute of Animal Physiology and Genetics, Czech Academy of Sciences, Veveří 97, 602 00 Brno, Czech Republic; 6 Faculty of Technology, Tomas Bata University in Zlín, Vavrečkova 5669, 760 01 Zlín, Czech Republic

**Keywords:** Tissues, Biomedical materials, Tissue engineering, Biomedical engineering

## Abstract

Polyetheretherketone (PEEK) is considered as an excellent biomaterial for bone grafting and connective tissue replacement. The clinical potential is, however, limited by its bioinertness, poor osteoconduction, and weak antibacterial activity. These disadvantages can be overcome by introducing suitable additives to produce mineral-polymer composites or coatings. In this work, a PEEK-based bioactive composite has been obtained by blending the polymer with magnesium phosphate (Mg_3_(PO_4_)_2_) particles in amounts ranging from 1 to 10 wt.% using the hot press technique. The obtained composite exhibited improved mechanical and physical properties, above the lower limits set for bone engineering applications. The tested grafts were found to not induce cytotoxicity. The presence of magnesium phosphate induced the mineralisation process with no adverse effects on the expression of the marker crucial for osteoblastic differentiation. The most promising results were observed in the grafts containing 1 wt.% of magnesium phosphate embedded within the PEEK matrix. The improved bioactivity of grafts, together with suitable physical–chemical and mechanical properties, indicate this composite as a promising orthopaedic implant material.

## Introduction

Polyetheretherketone (PEEK) is a high-performance, high-temperature thermoplastic material belonging to the polyaryletherketone group. It is used as a bioactive implant material in bone tissue engineering^1^, dentistry^2–4^, knee implants^5^, spine implants^6^, hip replacement^7^, and anterior plate fixation^8,9^. The degree of crystallinity varies depending on the thermal history of the polymer (specifically the manner in which the final product is cooled to room temperature), and it may range from 0% (i.e. amorphous form) to about 40% (semicrystalline polymer) when cooled down very slowly^10^. In terms of mechanical performance, PEEK is characterised by high creep resistance at temperatures below its glass transition point (145 °C)^11^.

The results of extensive in vitro tests based on long-term contact with cell cultures, as well as numerous in vivo studies, have confirmed that materials based on PEEK are not only cytocompatible but also compatible with living tissue. No mutagenic or carcinogenic effects or other adverse effects of this polymer have been reported^12^. However, its hydrophobic nature is limiting cell adhesion and, because it can be considered, in the biological sense, essentially inert, many efforts are devoted to improving its bioactivity. To this aim, mineral-polymer composites or coatings with calcium phosphates (hydroxyapatite (HA), tricalcium phosphate), phases already employed in the clinical practice, have been proposed^13–16^. Although the adhesion to the bone tissue improved significantly, with an increase in the modulus of elasticity, the strength and toughness of the composites dropped because of poor polymer adhesion to the phosphate particles^17^. The literature reports PEEK-HA composites with HA content up to 40%, but also PEEK surface modifications by plasma application, adopting various surface structuring metals, such as titanium and Ca-P-based compounds^18^. In fact, HA ceramic coatings are one of the most frequently proposed solutions. All in all, there is still insufficient information on aspects such as the role played by the type of mineral additive and its amount in determining the properties of the composites. HA also shows disadvantages in its application, due to its great fragility^19^, low surface area and porosity issues leading to a comparatively limited sorption capacity^20^. In this respect, other phosphate-based minerals (e.g. brushite (CaHPO_4_⋅2H_2_O), monetite (CaHPO_4_), struvite MgNH_4_PO_4_⋅6H_2_O, farringtonite Mg_3_(PO_4_)_2_) are considered promising^21–23^.

Magnesium compounds have been reported to behave favourably in bioactive composite materials. In fact, magnesium ions (Mg^2+^) are the fourth most abundant ions in mammals and are involved in several cellular functions. Osteogenic proliferation and differentiation were found to be enhanced in Mg-doped Ca-P-based materials^24^. Recently, amorphous magnesium phosphate, synthesized through the wet method, has been proposed as a component for a PEEK composite material^25^. The composite, produced as a filament, in view of employing it for 3D printed bioapplications, exhibited enhanced bioactivity and increased osteoblast proliferation. The obtained phosphate was a hydrated compound exhibiting a weight loss of approximately 15 wt.% during the process of filament formation (at temperatures > 350 °C).

In this work, the anhydrous Mg compound farringtonite (MP), was synthesised and mixed with PEEK in the amount of 1, 5 and 10 wt.%, to obtain a MP-PEEK composite which has been shaped using conventional processing (i.e. injection moulding). Although MP has been already employed in some Mg-based bio-cements^26–29^, this work is the first report of its use in the formulation of a bioactive MP-PEEK composite that can be used as feedstock for 3D printed bone grafting. The obtained samples were characterised in terms of surface characteristics, surface and volume distribution of the mineral, and mechanical properties. The cytocompatibility and bioactivity of the composite were evaluated in comparison to that of neat PEEK through cytotoxicity, osteoblast proliferation, and differentiation tests. The combination of these evaluation tests is important as it not only reveals the absence of adverse reactions but also allows to describe the bioactivity in terms of the effect on more advanced cell characteristics. The novelty of this study also resides in the application of a manually hot-pressed mixture, which is a simple and reproducible technological solution for the preparation of grafts, as well as in the integrated approach to the cytocompatibility evaluation aimed at revealing the impact on osteoinduction.

## Materials and methods

### Raw materials

MP was synthesised from a powder mixture of analytical grade MgHPO_4_⋅3H_2_O and Mg(OH)_2_ in the molar ratio of 2:1, which was sintered at 1150 °C for 4 h in a laboratory furnace^30^. The sintered material was ground by hand in an agate mortar to pass through a 63 µm sieve.

PEEK was obtained from Sigma Aldrich (USA) in the form of 6 mm granulate.

### Cell line and cultivation

*A cell line of mouse embryonic fibroblast (ATCC CRL-1658 NIH/3T3, USA) was used for cytotoxicity determination*. The medium for NIH/3T3 was formulated by ATCC as follows: Dulbecco's Modified Eagle's Medium (PAA Laboratories GmbH, xAUS) containing 10% of calf serum (BioSera, FR) and 100 U mL^−1^ Penicillin/Streptomycin (GE Healthcare HyClone, UK), was employed as the culture medium. The cells were incubated at 37 °C in 5% CO_2_ in humidified air.

*For osteoblast proliferation and differentiation*, a mouse osteoblastic precursors cell line, MC3T3-E1, was obtained from the European Collection of Cell Culture (c.n. 99072810). The proliferation conditions were obtained by cultivating of the cells in MEM Alpha medium (Gibco, USA) enriched by 10% FBS (Sigma Aldrich, USA) and penicillin/streptomycin (100 U mL^−1^, 100 µg mL^−1^, BioSera, FR). The differentiation conditions were adjusted by cultivating the cells in a differentiation medium prepared as described above but with the addition of 10 mM β-glycerolphosphate (βGP, Sigma Aldrich, USA) and 50 µg mL^−1^ ascorbic acid (AA, Sigma Aldrich, USA). Passages 3–7 were used for the experiments.

### Preparation of the grafts

The composites were prepared by mixing MP powder with PEEK melt using a microextrusion machine (Xplore, USA) at 380 °C. After 4 min of homogenisation, the mixtures were pressed at 380 °C for 3 min by the manual hot press into 1 mm thick sheets and cooled using a cooling device providing a constant temperature of 50 °C to constant cooling for 10 min. Subsequently, the samples in the form of discs with a diameter of 2.5 cm were punched out. Samples were labelled according to the percentage by weight of MP (0, 1, 5 and 10 wt. %), as follows: PEEK_0, PEEK_1, PEEK_5 and PEEK_10.

### Characterisation of the raw materials

A Bruker D8 Advance diffractometer in Bragg–Brentano *θ–θ* geometry, operated at 40 kV and 40 mA was employed to collect X-ray powder diffraction (XRPD) data from the synthesised MP powder in the angular range 10–60° 2*θ* adopting Cu Kα radiation (λ = 1.5418 Å).

The particle mean diameter (D) of the MP particles was obtained by laser granulometry adopting a CILAS 1090 (Orleans, FR) instrument, by dispersing the powder in isopropyl alcohol.

Brunauer–Emmett–Teller (BET) specific surface area of the powder was measured using an instrument ASAP 2020 (Micromeritics, Norcross, GA, USA).

The microstructure of the MP powder was observed using a scanning electron microscope (SEM) Quanta 450 FEG (FEI, CZ) in secondary electron mode at 20 kV accelerating voltage on a sample sputtered with a 7 nm thick gold film to reduce charging effects.

The spectra of the raw materials were measured on a Nicolet iZ10 secondary module (Thermo Fisher Scientific Inc., USA) equipped with diamond ATR crystal covering the range 4000–525 cm^−1^ at a resolution of 4 cm^−1^.

The thermal decomposition of PEEK granulate was determined by a combination of Differential thermal analysis (DTA)-Thermogravimetric analysis (TGA) using an STA 504 thermal analyser (TA Instruments, DE). This method was used for real-time measurements of weight loss of the examined materials as a function of temperature. Measurements were performed under an N_2_ atmosphere at a heating rate of 20 °C min^−1^ in the 30–1000 °C temperature range. Samples were cut into small rectangular pieces weighing approximately 15 mg.

### Physical–chemical and mechanical characterisation of the grafts

Fourier transform infrared (FTIR) maps were acquired with a Nicolet iN10 FT-IR microscope (Thermo Fisher Scientific Inc., USA) in order to detect the spatial distribution of MP. The maps were obtained from the surfaces and cross-sections of all the samples over an area of 300 × 200 µm with a step size of 10 µm. Each spectrum was collected from a 10 × 10 µm area accumulating 64 scans with a spectral resolution of 8 cm^−1^ in the 4000–675 cm^−1^ spectral range.

A Keyence VHX-6000 confocal microscope (Keyence, Mechelen, BE) with a VHX-S600E free-angle observation system (Z-motorized) was used for the characterisation of samples in terms of surface roughness (S_a_: arithmetical mean roughness value and S_z_: mean roughness depth) was determined using ISO 25178^31^. Samples were characterised before and after osteoblast proliferation and differentiation tests. Samples were thoroughly cleaned with isopropyl alcohol after osteoblast proliferation and differentiation tests.

The size of the contact angle (CA) was measured to determine the wettability of the samples before and after osteoblast proliferation and differentiation tests. Samples were thoroughly cleaned with isopropyl alcohol after osteoblast proliferation and differentiation tests. The measurements were performed using a See System instrument (Advex Instrument, CZ), where five measurements were performed for each sample, and the mean and standard deviation were calculated. Water was chosen as the liquid, the drop size was 10 µL, and the reading of the size of the CA was taken after 10 s. The measurement was performed at room temperature (RT, 23 ± 2 °C).

Tensile strength tests were conducted on the clear samples with dimensions 1 × 2.5 × 0.01 cm by means of an Instron 1122 (Instron, USA) instrument at a maximum load of 5 kN at a speed of 5 mm·min^−1^. The results have been reported as an average of five replicate measurements.

The Brinell hardness measurements were obtained using a machine NEMESIS 9000 (INNOVATEST, NL) with a charge of 31.25 kgf for 10 s, and a minimum of 5 indentations were taken for each clear sample.

### Cytocompatibility evaluation

#### Grafts cytotoxicity

The test was performed in accordance with the ISO standard 10993-5^32^ Tested materials were extracted in a culture medium for 24 h at 37 °C with stirring. Extracts were prepared from sterilised samples (by ethanol for 1 h), thus eliminating the need for filtration of extracts. The parent extracts (100%) were then diluted in a culture medium to obtain a series of dilutions. The extracts were used for up to 24 h. The preparation of the extracts was conducted in accordance with the ISO standard 10993-1233, with a concentration of 3 cm^2^ mL^−1^ of media.

Mouse embryonic fibroblasts were seeded to pre-incubate in the 96-well plates (TPP, CH). The concentration of cells was 10^5^ cells mL^−1^. The extracts were diluted with a medium to obtain the following concentrations: 100, 75 and 50% of the parent extract. The medium was removed and replaced by individual extracts. All assays were performed in quadruplets. Cell viability was determined by the MTT cell proliferation assay (Duchefa Biochemie, NL). The absorbance was measured at 570 nm with an Infinite M200 Pro NanoQuant instrument (Tecan, CH) and the reference wavelength was adjusted to 690 nm. The statistical significance of the results was determined by one-way ANOVA with post hoc Tukey's Multiple comparison test (P < 0.05).

#### Grafts bioactivity; osteoblast proliferation and differentiation

The osteoblasts were suspended in Trypsin (0.25%, Gibco, USA), and subsequently centrifuged to remove the enzyme, after which they were re-suspended in proliferation or differentiation media for further analyses. The PEEK composite discs were added into 6 well plates (TPP, CH) and washed with sterile 1 × Phosphate Buffered Saline (PBS, BioSera, FR). 500 µL of cell suspension (10^6^ cells mL^−1^) was added to the surface of the composite discs and allowed to adhere in the incubator for 2 h. A control cell sample was seeded onto a Petri dish without the disc. The cells were then covered with fresh medium and cultured for several days. The medium was changed every 3 days.

### RNA isolation and qPCR

The cultured cells were harvested into 350 µL RLT lysis buffer (Qiagen, Valencia, CA) with β-mercaptoethanol (Sigma-Aldrich, USA) after 6 days of cultivation in proliferating media. Total RNA was extracted from the MC3T3-E1 cells using RNeasy Mini Kit (Qiagen, DE). RNA concentration and purity were assessed using a NanoDrop, and cDNA was synthesised using the reverse Master Mix (Generi Biotech, CZ). For cDNA synthesis, 500 ng of RNA was used, and biological replicates of each sample were diluted to a concentration of 100 ng/μl before performing a qPCR reaction. The qPCR was performed in a 10 μL final reaction volume containing the one-step Ideal PCR Master Mix (Generi Biotech, CZ) using the Light-Cycler 96 (Roche, CH) with preheating to 95 °C for 10 min. This was followed by 40 cycles of 95 °C/15 s and 62.5 °C/1 min with mRNA probes for *Col1a1, PCNA, Runx2, Spp1* (Mouse *Col1a1*, Mm00801666_g1; *PCNA*, Mm00448100_m1; *Runx2*, Mm00501584_m1; *Spp1,* Mm00436767_m1; TaqMan Gene expression Assay, Thermo Fisher Scientific, UK). Expression levels were calculated using the ΔΔCT method, with normalization against *actin* RNA levels (mouse *Actb*, Mm02619580_g1, TaqMan Gene Expression Assay, Thermo Fisher Scientific, UK). Three biological replicates were performed at each developmental stage.

### Statistical analysis

All results were expressed as mean ± standard deviations (SD) of three samples for each time point and compared using one-way ANOVA (One-way analysis of variance, Dunnett test: Compare all pairs of columns, Significant level = 0.05). Differences were considered as significant at p < 0.05 indicated by the * symbol.

### Cell staining

To ascertain the impact of PEEK on osteoblast behaviour, three commonly used cell staining techniques were employed:

*Mayer Haematoxylin was used for cell visualisation.* Cells cultivated in a proliferation medium for 10 days were fixed and stained with Haematoxylin (DiaPath, IT).

*Levels of alkaline phosphatase were observed by staining ALP.* Cells cultivated in a differentiation medium for 14 days were fixed and stained with 300 μL of Fast blue mixture containing 4 mg of naphthol AS-TR phosphate disodium salt (Sigma Aldrich, USA) in 150 μL of N,N-dimethylformamide (Fluka Chemicals, CH) and 12 mg of Fast blue BB Salt hemi(zinc chloride) salt (Sigma Aldrich, USA) in 15 mL of 0.1 M Tris–HCl buffer (pH 9.6) for 4 h in the dark.

*Von Kossa staining was used to detect the presence of calcium deposits.* Cells cultivated in differentiation media for 14 days were fixed and washed with distilled water. The water was removed, a 2% silver nitrate solution was added, and the plate was exposed to sunlight for 60 min, after which the plate was rinsed with distilled water (dH_2_O). Subsequently, sodium thiosulfate (5%) was added for 10 min, the plates were then rinsed in dH_2_O, and nuclear red was added for 5 min. Finally, the plates were washed with dH_2_O.

## Results and discussion

### Characteristics of raw materials correspond to pure materials

**Figure 1:**
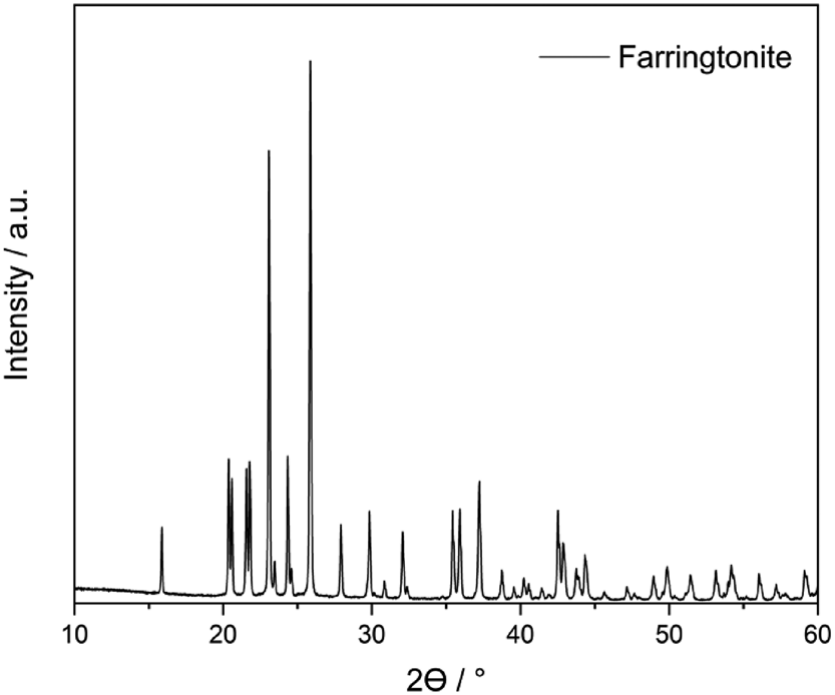
XRPD pattern of MP.

**Figure 2:**
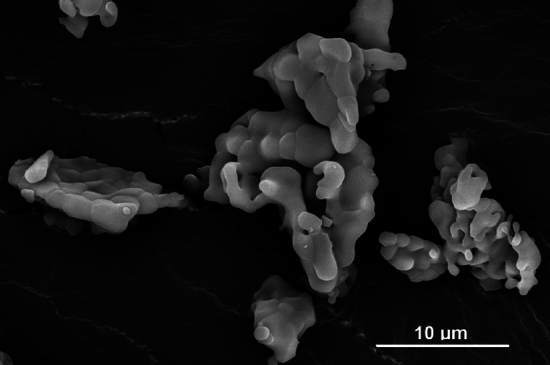
SEM observation of MP structure.

The XRPD analysis confirmed the purity of the synthesised MP **(**Fig. 1**)**, as all diffraction peaks pertained to the mineral^33,34^. The sintered MP particle size characteristics were as follows: D_10_ = 5.09 µm, D_50_ = 23.42 µm and D_90_ = 50.27 µm. The BET specific surface area was found to be 0.575 ± 0.05 m^2^ g^−1^. These results are in line with those from MP powder proposed for bio-applications^35^. Under the electron microscope, the powder particles exhibited irregular shapes, which can be attributed to the coalescence of smaller subspherical grains (Fig. 2). This observation is in agreement with the literature^34^. The coalescence is a typical effect of sintering. The shape of the grains can significantly influence the physical–mechanical properties of the resulting polymer material. A shape different than spherical may induce a more pronounced anisotropy^36^.

**Figure 3:**
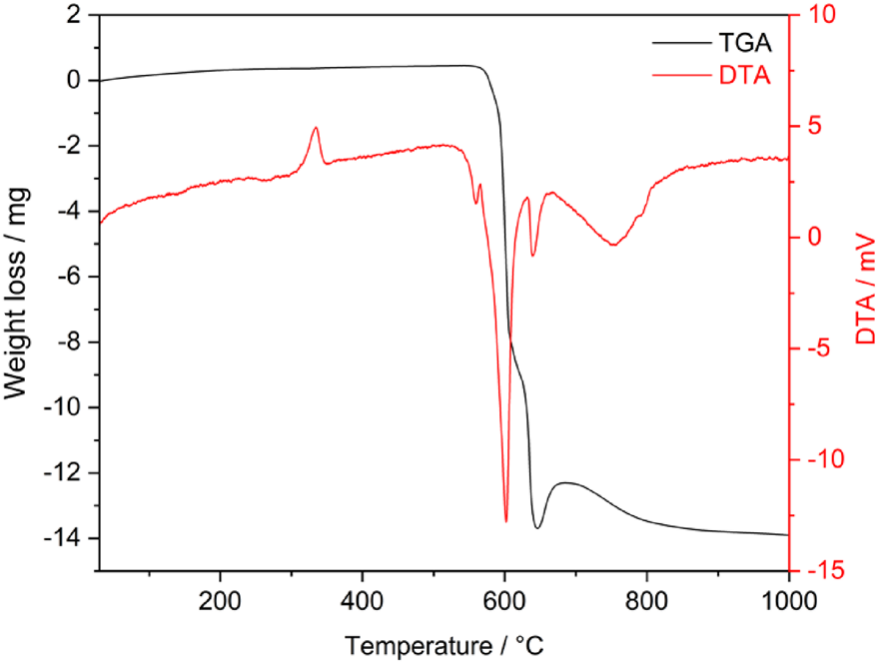
TGA-DTA curve for the employed PEEK.

The thermal characteristics of the PEEK polymer were obtained from the DTA-TGA measurements graphically illustrated in Fig. 3. The onset of degradation was found at 591.9 °C, which corresponds to the formation of a charred structure. These results are in good agreement with the literature^37^. The DTA peak at 334.3 °C corresponds to the melting point of the polymer. Temperatures in excess of 400 °C are not recommended for conventional processing methods to prevent degradation and cross-linking of the structure^38^. Based on the obtained results, a processing temperature of 380 °C was chosen.

### The physical–chemical and mechanical properties of grafts are suitable for bone tissue engineering

The grafts were characterised in terms of surface properties both before and after the osteoblast proliferation and differentiation test, because surface roughness and CA are usually related to bone cell adhesion^39^. The CA of neat PEEK resulted 75.5° ± 2.3° (Table 1), which is in agreement with some results from the literature^40^, although different values can be found, in reason of several variables related to the state of the surface ^41^. For example, contact angle as high as 100° has been recently reported in 3D printed neat PEEK scaffolds^42^. In the studied samples, the increase in MP content increased the CA up to 93.7° ± 0.1°.

**Table 1:** Characterisation of the sample surface before and after osteoblast proliferation and differentiation tests.

Sample	Contact angle (°)		Surface roughness			
	Before	After	Before		After	
			S_a_ (µm)	S_z_ (µm)	S_a_ (µm)	S_z_ (µm)
PEEK_0	75.5 ± 2.3	72.2 ± 3.1	1.0 ± 0.2	15.6 ± 0.2	0.9 ± 0.1	7.8 ± 0.1
PEEK_1	85.5 ± 1.5	72.7 ± 6.6	0.9 ± 0.1	13.2 ± 0.1	1.3 ± 0.2	5.7 ± 0.3
PEEK_5	88.4 ± 3.1	77.2 ± 3.6	1.1 ± 0.1	12.2 ± 0.1	0.6 ± 0.1	6.7 ± 0.2
PEEK_10	93.7 ± 0.1	71.4 ± 4.1	1.2 ± 0.2	17.9 ± 0.2	0.9 ± 0.1	7.6 ± 0.1

When turning to the samples after the osteoblast proliferation and differentiation test, the material appeared to be more hydrophilic (CA < 77°) without any clear dependence from the mineral content of the composite. It is known that wettability should be related to the surface roughness^41^, therefore, it might be argued that the lower CA measured in the samples after the biotests is the result of its smoother surface. The lower roughness parameter Sz in these samples could be ascribed to the presence of residual proteins bound to the surface^43^.

No clear explanation of the above results is available to the authors. In fact, the presence of fillers has been documented to impact on wettability; in composites, CA can be calculated by applying the equation developed by Cassie and Baxter, provided same surface parameters^44^. With the introduction of a filler with lower CA, this equation predicts a decrease^45^. This is what we should expect in our case, since the parameters of surface roughness are not changing appreciably with amount of MP and the measurement on a pressed pellet of MP indicated a CA of 43°, much lower than PEEK. Nonetheless, an opposite trend in function of MP content is observed in the samples before biotests, whereas no trend is observed after the tests.

Therefore, it will be necessary to rely on the results of biotests for the evaluation of the role of MP in the PEEK composites. Osteoblast proliferation and differentiation have been reported to be influenced by surface topography, as well as by material formulation (e.g., presence of fillers). In fact, previous studies indicated that injection-moulded PEEK exhibited better initial cell adhesion^46^. This seemed related to its smoother surface, although other characteristics may play a role when compared to the rougher machined surfaces. For example, the surface of the produced PEEK is typically covered with a thin polymer layer formerly in contact with the mould. This skin is different from the bulk and masks local variations in the material, especially the presence of amorphous and crystalline polymer domains^11^. As mentioned in the introduction, the degree of crystallinity of PEEK changes in function of the processing parameters, especially the thermal history.

**Figure 4:**
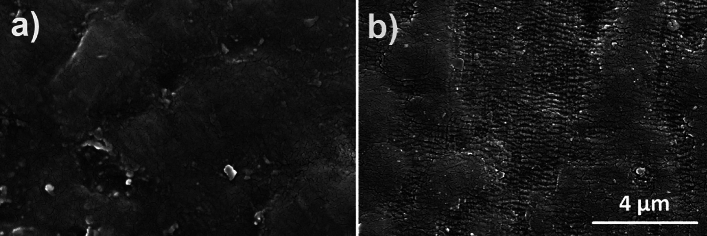
SEM observation of the selected samples after osteoblast proliferation and differentiation tests, where (**a**) PEEK_0 and (**b**) PEEK_10.

When considering the appearance of the graft surface, prior to biotesting, the samples exhibited a uniform surface finish that was consistent with what is usually observed when conventional plastics processing technology is employed. Figure 4 illustrates some surface modification detected after osteoblast proliferation and differentiation tests in the sample PEEK_10. Despite the similar indicators of surface topography (Table 1), this could be tentatively ascribed to the partial degradation of MP. In fact, degradation in vivo has been reported to lead to the formation of a CaP-based layer^47^.

In this respect, besides the assessment of biocompatibility *in vivo*^48^, the degradation behaviour of compounds in the MgO-P_2_O_5_ binary system, constituents of biocements, may provide clues to interpret our results. All in all, they evidenced a superior degradation potential with respect to Ca-phosphates ^29^. Indeed, compared to the hydrated magnesium phosphates phases, farringtonite persisted for longer times in the physiological environment, because of its lower solubility^21^. It was shown that in vivo degradation of the magnesium phosphate compounds in time leads to the precipitation of calcium phosphate whitlockite (Ca_3_(PO_4_)_2_). Implanted farringtonite scaffolds were observed to promote bone formation and remodelling, indicating the onset of an active resorption mechanism^47^. These results were confirmed in an animal model for polymer-modified farringtonite scaffolds^49^. At the same time, no potential adverse effects of the release of Mg^2+^ ions following farringtonite degradation (e.g., influence on homeostasis, inhibition of apatite crystal growth) have been reported. They are considered absent^29^, although the literature is scarce, being this of Mg-based biomaterials a relatively young field of research.

Therefore, bioresorption of the MP particles at the PEEK surface, occurring in vivo, might be thought to have positive effects for bone regeneration and also favour adhesion to the pristine PEEK surface by leaving surface irregularities.

**Figure 5:**
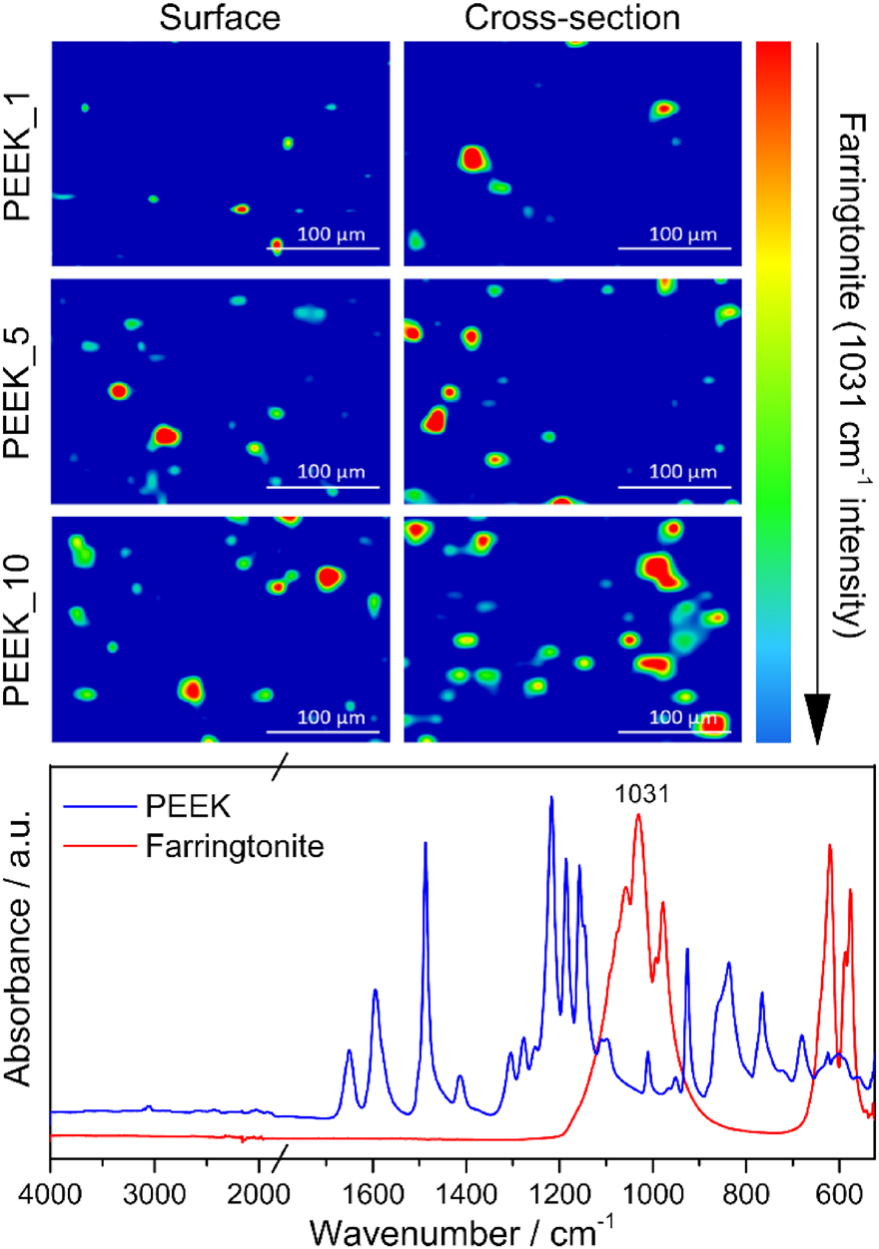
FTIR microscope mapping illustrating the distribution of MP (green to red) in the PEEK matrix (blue) on the surface of the samples (left) and the cross-section (right). FTIR spectra of the two phases are shown at the bottom part of the figure.

The distribution of the mineral on the surface of the samples and in cross-section is well-illustrated by the FTIR maps in Fig. 5. The intensity of the most intense PO_4_^3−^ stretching vibration at 1031 cm^−1^ was used to identify MP in the composites^50^. The detection of the spatial distribution of MP, was possible thanks to the compatibility of the spatial resolution of the maps with the grain size of MP. The obtained maps evidenced a relatively homogeneous distribution of the additive in both cases, with some limited tendency to form aggregates of particles at the highest concentration.

The results of the mechanical tensile tests carried out on the composites are summarised in Table 2. While the values for neat PEEK (PEEK_0) are in line with those reported in the literature^51^, the composites were found to perform better, with the Young's modulus of elasticity increasing towards the high range of the elastic modulus of human cancellous bone (1.3–7.8 GPa^52,53^). A clear trend of increasing strength and maximum elongation at failure with content in MP, is observed.

**Table 2:** Mechanical test results.

Sample	Brinell hardness test	Tensile test		
		Young modulus (GPa)	Tensile strength (MPa)	Elongation (%)
PEEK_0	18.96 ± 0.40	5.8 ± 0.43	67.1 ± 1.2	16.9 ± 5.1
PEEK_1	18.72 ± 0.14	6.2 ± 0.51	76.3 ± 0.5	18.5 ± 2.8
PEEK_5	18.79 ± 0.08	7.7 ± 0.67	82.5 ± 7.3	19.2 ± 2.0
PEEK_10	18.48 ± 0.04	7.9 ± 0.40	81.1 ± 6.1	22.8 ± 1.9

Similar effects have been documented for other polymer reinforced composites, in function of the size and shape of the filler particles^54,55^. Conversely, Brinell hardness measurements did not evidence significant changes.

### Grafts do not induce cytotoxicity

**Figure 6:**
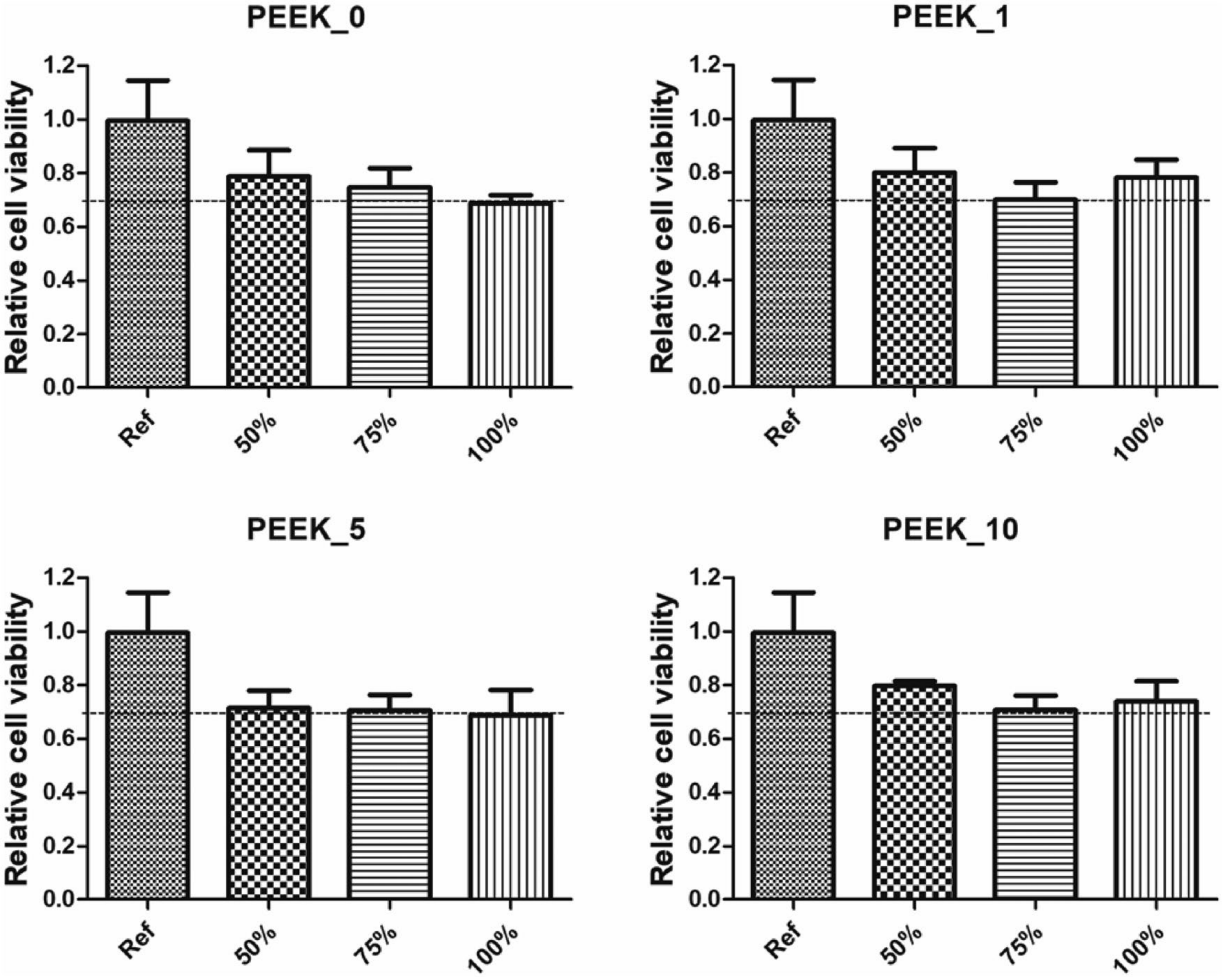
The cytotoxicity of PEEK disks was evaluated by a decrease in cell viability of NIH/3T3 cells compared to the reference. The graphs show the averages of relative cell viability with standard deviation. Dashed lines mark the cytotoxicity threshold.

Cytocompatibility is a crucial property of any biomaterial^56,57^. Figure 6 illustrates the results of cytotoxicity tests expressed as a reduction in cell viability in percentage when compared to cells cultivated in a medium without the tested extract of the sample. No significant statistical difference was observed between the neat PEEK and the composites, but statistical analysis showed a difference between the reference and individual extracts for all samples except the 50% extract of PEEK_0, but no clear differences between the samples. From the point of view of interpreting the results, more important is the evaluation of the cytotoxicity rate according to ISO 10993-5, which sets a threshold of 70% relative to the reference. In several cases, relative cell viability approached the cytotoxicity threshold (70%). However, the viability never decreased below this level. It can be concluded that none of the composites induced cytotoxicity.

While cytotoxicity is considered as a general prerequisite for any biomaterial, the interaction with cells of the intended target tissue can provide more specific information about cytocompatibility and bioactivity. The ability of osteoblasts to adhere, proliferate and differentiate on the surface of the composites was thus further tested.

### The bioactivity of grafts correlates to the amount of MP

**Figure 7:**
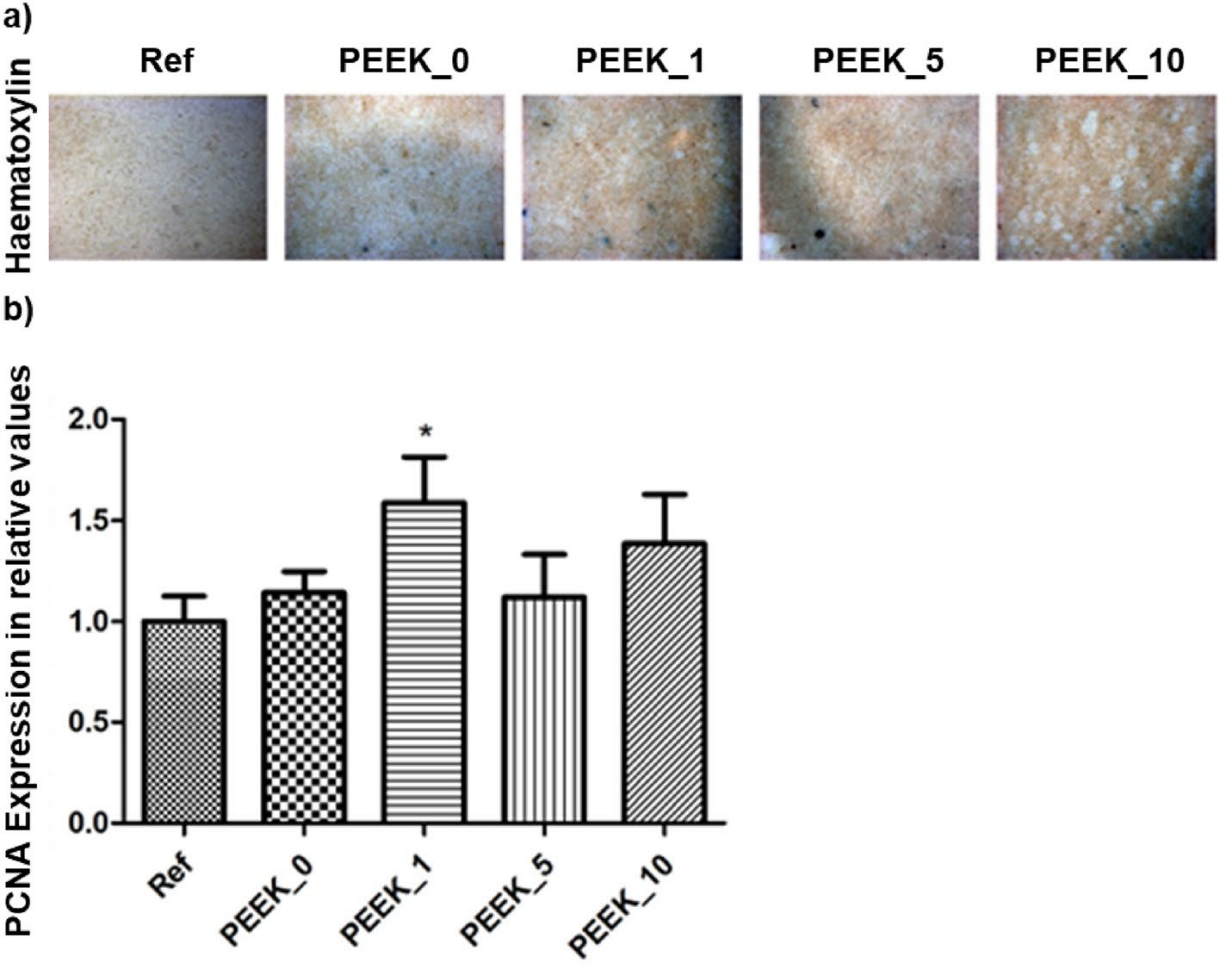
PEEK modification shows compatibility with calvaria pre-osteoblasts. The cells cultivated at the modification surface for 10 days displayed natural adhesion and proliferation as visualised by haematoxylin staining, magnification 20×. The proliferation marker PCNA expression levels were compared with reference cells cultivated without PEEK disc. The graph shows the expression (mRNA) of PCNA compared to reference. Data are expressed as mean ± SD (n—4 per group) and statistically significant difference is highlighted (Dunnett's multiple comparisons test *p < 0.05).

Appropriate cell adhesion and proliferation is the prerequisite for bioactivity experiments. The micrographs of the cell proliferation on the material surfaces are shown in Fig. 7a. The cells cultured on the PEEK disc did not exhibit an alteration of proliferation with an increasing concentration of MP (Fig. 7b). Moreover, the qPCR analysis of the cells cultivated on PEEK discs after 6 days confirmed the suitability of the discs for osteoblast cell proliferation (Fig. 7b). Significant changes in PCNA expression, and thus the proliferation of cells, were observed only in the case of grafts containing 1% of MP, which thus seems to be the most promising for in vivo testing. The analysis revealed an increased level of proliferation on the disc in the case of 1% modification (* indicates p ≤ 0.05, Dunnett's multiple comparisons test).

The absence of cytotoxicity and the ability of osteoblasts to adhere and proliferate on the grafts in a manner comparable to reference materials confirm the cytocompatibility of the grafts. However, successful implantation, also anticipates adequate cell physiology. Therefore, a qualitative analysis of calcium deposition (confirmed by Von Kossa staining) and cell differentiation (confirmed by ALP activity) was performed.

**Figure 8:**
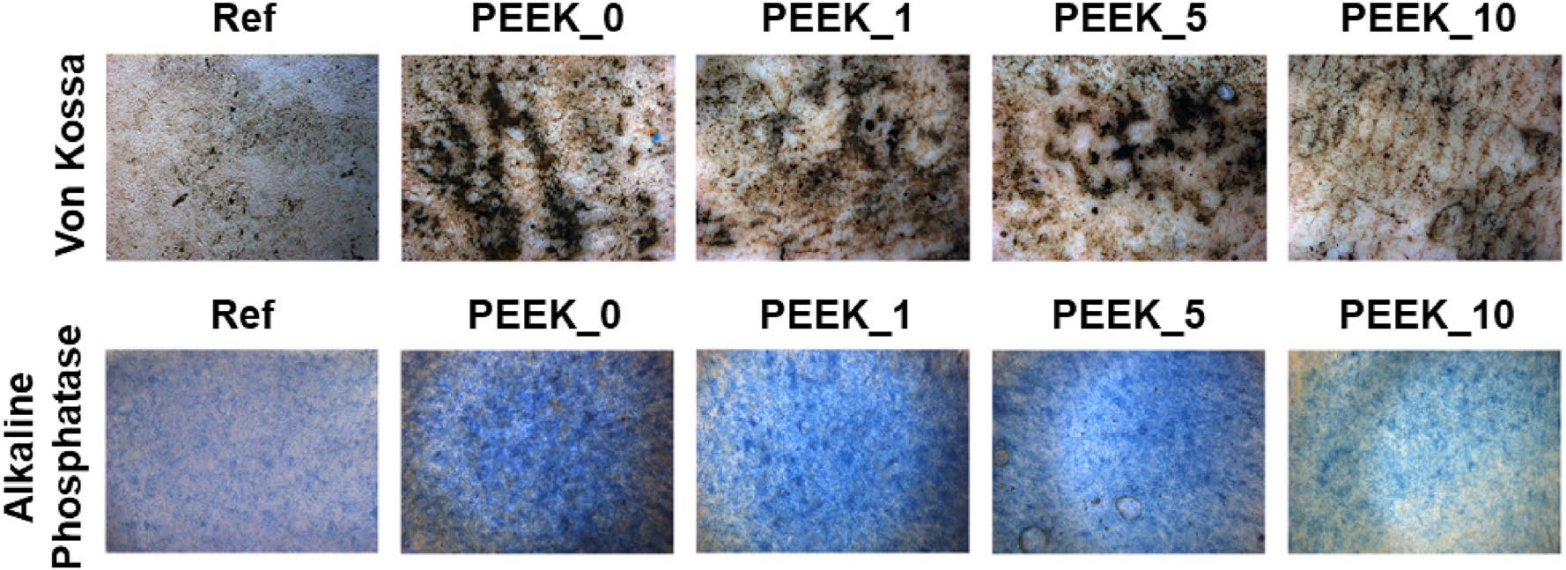
Staining of the mineralisation and alkaline phosphatase activity in MC3T3-E1 cells cultivated on the PEEK and control cells, magnification 20×. The increased Ca^2+^ depositions (black dots) were observed in the cells cultivated on the PEEK surface. Increased ALP activity was detected in the case of 0%, 1% and 5% modification. Positive cells are blue.

The results of the calcium deposition and ALP activity are presented in Fig. 8. The Von Kossa method revealed increased mineralisation levels (black) of MC3T3-E1 pre-osteoblasts cultivated on the PEEK surface compared with cells cultivated in a Petri dish after 14 days of cultivation. The level of Ca^2+^ was decreased in the 10% PEEK modification (Fig. 8). In Fig. 8, an abundance of ALP staining cells (in blue) was observed on the PEEK disks, and these spots were denser than in the control samples. The decreased level of ALP-positive cells was observed in the case of the 10% modification as well as in the Ca^2+^ mineralisation analysis.

**Figure 9:**
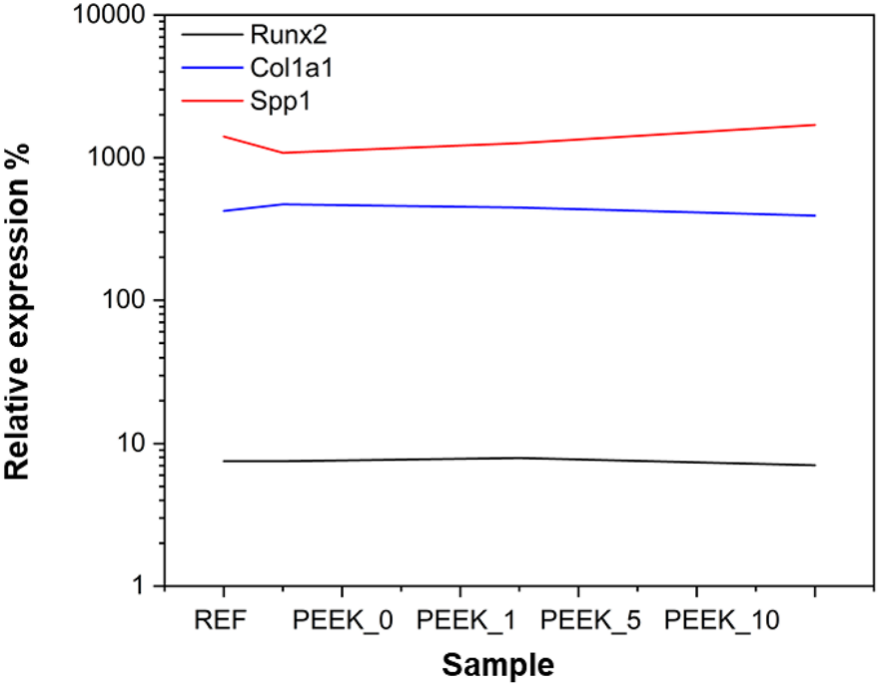
Comparison of relative expression levels of investigated molecules. The graph shows the relative expression levels of *mRNA*s corresponding to Runx2, Col1a1, and Spp1 in control, 0% (PEEK_0), 1% (PEEK_1), 5% (PEEK_5) and 10% (PEEK_10) PEEK. No significant differences in the expression of the selected markers were observed with respect to the reference.

To compare the analysis of staining for mineralisation and osteogenic potential of PEEK modification, the expression of certain osteogenic genes was compared to cells cultured simultaneously under different conditions on PEEK discs. The modifications of the PEEK do not significantly change the expression of early osteoblast marker *Runx2*, osteoblast marker *Col1a1* and differentiated osteoblast marker *Spp1* compared with cells cultivated on the standard dish*.* The relative expression of these genes to *Actin* in Fig. 9 shows that PEEK and its modification do not affect the osteoblastic differentiation. In summary, while Fig. 8 suggests enhanced bone formation with modified PEEK, Fig. 9 indicates that the gene expression related to osteoblastic differentiation is not significantly affected. The conclusion drawn is that PEEK and its modifications exhibit great properties for osteoblastic cell cultivation and have potential for connective tissue replacement, despite the observed variations in calcium deposition and ALP activity. Therefore, in the present case, the controversial results of surface characterization, pointing to a worsening of the properties, could not be considered predictive of the behaviour of the composites with respect to osteoblasts adhesion and proliferation.

## Conclusions

PEEK grafts have high potential in a wide range of dental, craniofacial, and orthopaedic applications, but its use is still limited to poor cell osteointegration. Here, we have shown that the modification of the PEEK with MP increases the possibility of using PEEK in orthopaedics thanks to their bioactivity. The physical–chemical and mechanical properties of both the unmodified and modified grafts are suitable for bone-related applications. Although the cytotoxicity test showed a non-significant declining trend in the viability of fibroblasts, the results with osteoblast showed an upward trend. The osteoblasts adhesion and proliferation on the graft surfaces is at least comparable to reference materials, and in the case of 1% of MP in the PEEK matrix, is even significantly better. The differentiation analysis showed that the presence of MP could induce the mineralisation process, and the modification does not affect the expression of the marker crucial for osteoblastic differentiation. By summarising our results, MP may contribute to the clinical treatment approach for bone defects and diseases based on PEEK.

## Data Availability

The datasets used and/or analysed during the current study available from the corresponding author on reasonable request.
